# Plasma cytokine and angiogenic factors associated with prognosis and therapeutic response to sunitinib vs everolimus in advanced non-clear cell renal cell carcinoma

**DOI:** 10.18632/oncotarget.15011

**Published:** 2017-02-02

**Authors:** Pavlos Msaouel, Amado J. Zurita, Shixia Huang, Eric Jonasch, Nizar M. Tannir

**Affiliations:** ^1^ Department of Genitourinary Medical Oncology, The University of Texas M.D. Anderson Cancer Center, Houston, TX, USA; ^2^ Dan L. Duncan Cancer Center & Department of Molecular and Cellular Biology and Alkek Center for Molecular Discovery, Baylor College of Medicine, Houston, TX, USA

**Keywords:** biomarkers, cytokines and angiogenic factors, everolimus, non-clear cell renal cell carcinoma, sunitinib

## Abstract

No biomarkers are available to predict relative clinical benefit from targeted therapies in patients with non-clear cell renal cell carcinoma (nccRCC). To identify candidate predictive markers, we investigated a set of cytokines and angiogenic factors (CAFs) in previously untreated patients with nccRCC participating in the phase II ESPN trial comparing first-line sunitinib to everolimus. Pre-treatment concentrations of 30 CAFs were measured in plasma from 37 patients treated with everolimus (n=16) or sunitinib (n=21), and associated with progression-free (PFS) and overall survival (OS) after adjusting for potential confounders. High (>median) concentrations of soluble glycoprotein 130 (sgp130) were predictive of a longer PFS with sunitinib compared with everolimus (HR = 0.30; 95% CI: 0.11-0.85; P = 0.024). Significantly shorter PFS was noted, independently of treatment arm, in patients with high (>median) levels of IL-8 (HR = 3.13; 95% CI: 1.41-6.92), IL-13 (HR = 3.36; 95% CI: 1.49-7.58), and soluble tumor necrosis factor receptor II (HR = 2.21; 95% CI: 1.04-4.72). High IL-8 levels were also associated with significantly shorter OS (HR = 3.55; 95% CI: 1.55-8.14). Thus, using CAF profiling we identified candidate prognostic and predictive circulating biomarkers that can be used to inform therapeutic decisions in nccRCC.

## INTRODUCTION

The non-clear cell types (nccRCC) account for approximately 25% of all renal cell carcinoma (RCC) cases [1, 2] and represent a genetically and histologically diverse group of cancers that, in descending order of prevalence, includes papillary, chromophobe, unclassified RCC, Xp11.2 translocation RCC, and other rare subtypes [2]. Agents targeting the vascular endothelial growth factor (VEGF) or the mechanistic target of rapamycin (mTOR) [3] are less effective against nccRCC compared with the more common clear-cell RCC (ccRCC) histology [4]. However, to date no other targetable pathways have been validated against nccRCC. To optimize therapeutic efficacy and help clinicians choose between upfront VEGF-directed agents vs mTOR inhibitors in nccRCC, predictive biomarkers need to be identified.

Molecular pathways associated with angiogenesis may be of relevance to a subset of nccRCC cases. Alterations of the von Hippel-Lindau (*VHL*) gene, a known hallmark of ccRCC, have been reported in approximately 16% of nccRCCs [5]. Furthermore, mutations in the hepatocyte growth factor (HGF) receptor *MET* are often found in patients with papillary RCC [6]. Both VHL and MET crosstalk with the VEGF pathway and play a key role in tumor cell proliferation and angiogenesis. Hyperactivation of mTOR is also found in a subset of nccRCCs, providing rationale for therapeutic inhibition of this signaling pathway [6]. Further supporting the molecular heterogeneity of nccRCCs, we recently profiled cytokines and angiogenic factors (CAF) in plasma of nccRCC patients and found that whereas some express high levels of pro-angiogenic and inflammatory tumorigenic markers, others are characterized by relatively low concentrations of these CAFs [7]. We further found that elevated pre-treatment levels of CAFs including interleukin 8 (IL-8), tumor necrosis factor alpha (TNFα), soluble tumor necrosis factor receptor I (sTNF-RI), and soluble vascular endothelial growth factor receptor-2 (sVEGF-R2) were associated with worse prognosis in patients with nccRCC [7].

Sunitinib is a multitargeted antiangiogenic tyrosine kinase inhibitor (TKI) of vascular endothelial growth factor receptors (VEGFRs) that prolongs the progression-free survival (PFS) survival of patients with metastatic RCC, and has clinical activity in nccRCC [8, 9]. In addition, prospective trial data have shown that subgroups of patients with nccRCC may benefit from treatment with the mTOR inhibitor everolimus [10, 11]. The multicenter, open-label, randomized phase II ESPN trial (Everolimus versus Sunitinib Prospective Evaluation in Metastatic Non-clear cell Renal Cell Carcinoma) compared everolimus to sunitinib as frontline treatments in advanced nccRCC. It did not meet its primary endpoint of finding an improvement of PFS with everolimus over sunitinib when these agents were given as first-line therapy, although the median PFS was numerically longer with sunitinib than with everolimus (6.1 months versus 4.1 months) [12]. Following disease progression, patients crossed over to the drug they did not receive upfront. Overall survival (OS) did not differ between the patients who received upfront everolimus vs sunitinib. Exploratory subgroup analyses according to histology also did not reveal any differences in outcomes between the two therapies [12].

In this work, we profiled a broad set of plasma CAFs from patients enrolled in the ESPN trial to: (i) confirm and expand in an independent patient group our previously identified prognostic CAF candidates in patients with nccRCC, (ii) screen for CAFs predictive of differential benefit from sunitinib versus everolimus before any treatment is started, and (iii) identify common CAF profiles across the different nccRCC subtypes associated with distinct responses to therapy.

## RESULTS

### Baseline characteristics, CAF levels and patient outcomes

Baseline CAF levels and clinical information were analyzed from 37 patients in the ESPN trial. Table 1 and the supplementary table show that the demographics, baseline characteristics and CAF levels of each treatment group were well-balanced with the exceptions of SCF and sVEGF-R2, which were significantly higher in patients treated with sunitinib. After a median follow-up time of 22.6 months (range, 2.2 - 68), 28/37 (75.7%) had died and only 2 (5.4%) were alive without progression at the last follow-up visit. There was no statistically significant difference (p=0.101) in the median PFS for the everolimus group (2.7 months) compared with the sunitinib group (5.8 months). Similarly, the median OS for the everolimus group was 13.6 months vs. 11.4 months for the sunitinib group (p=0.32).

**Table 1:** Baseline characteristics of each treatment group

	Everolimus (n=16)	Sunitinib (n=21)	p value
**Age^a^**	46.5 (42.0 – 62.5)	55 (50 - 62)	0.365^c^
**Gender^b^**			
Male	11 (68.8%)	11 (52.4%)	0.50^d^
Female	5 (31.2%)	10 (47.6%)	
**Race^b^**			
Caucasian	15 (93.7%)	16 (76.2%)	0.206^d^
Other	1 (6.3%)	5 (23.8%)	
**ECOG performance status^b^**			
0	5 (31.2%)	9 (42.9%)	0.515^d^
1	11 (68.8%)	12 (57.1%)	
**MSKCC risk group^b^**			
Favorable	0	3 (14.3%)	0.153^d^
Intermediate	15 (93.7%)	18 (85.7%)	
Poor	1 (6.3%)	0	
**IMDC risk group^b^**			
Favorable	0	3 (14.3%)	0.265^d^
Intermediate	15 (93.7%)	15 (71.4%)	
Poor	1 (6.3%)	3 (14.3%)	
**Prior nephrectomy^b^**			
No	2 (12.5%)	9 (42.9%)	0.071^d^
Yes	14 (87.5%)	12 (57.1%)	
**Histology^b^**			
Papillary	5 (31.3%)	6 (28.6%)	0.469^d^
Chromophobe	2 (12.5%)	4 (19.0%)	
Clear cell with sarcomatoid features	5 (31.3%)	6 (28.6%)	
Translocation	0	3 (14.3%)	
Unclassified	4 (25.0%)	2 (9.5%)	

^a^Years; data are given as median values (interquartile range)

^b^Categorical variable; data in parentheses represent the percentage of each group

^c^Mann–Whitney U test

^d^Fisher's exact test

### Prognostic and predictive value of pretreatment CAF levels

**Figure 1:**
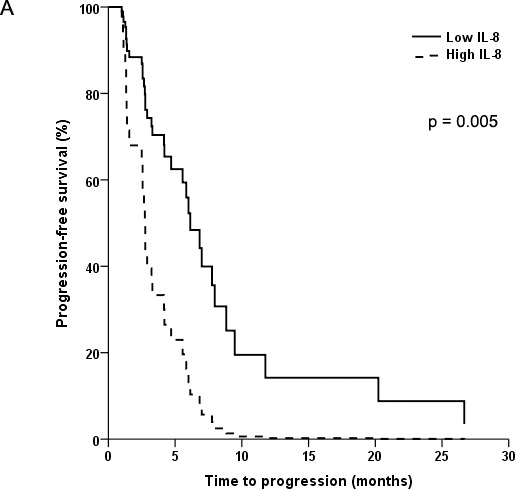

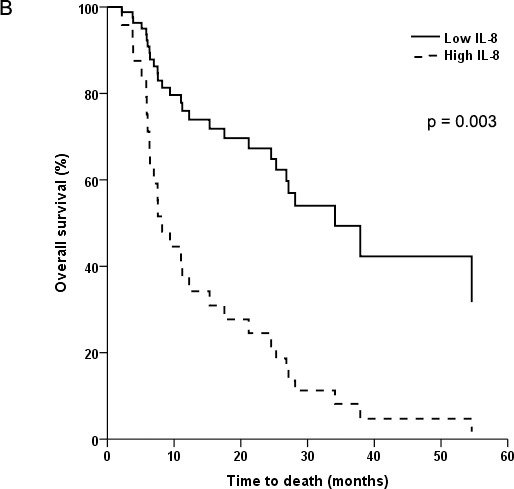

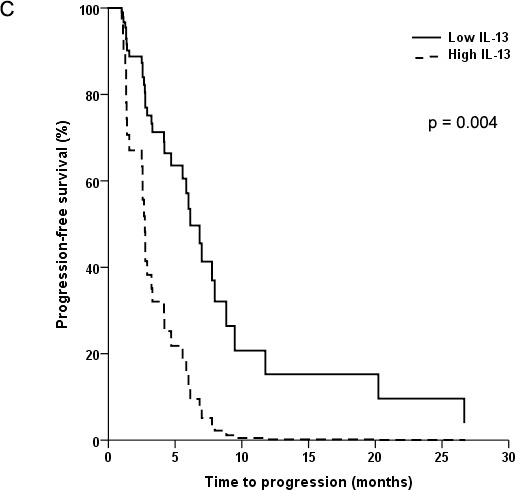

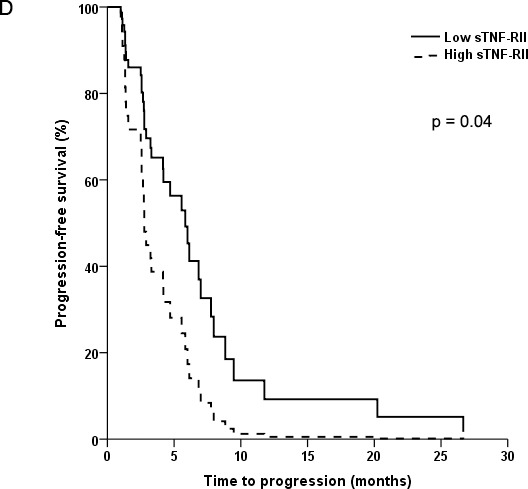
Kaplan-Meier plots of progression-free survival (PFS) and overall survival (OS) in patients with advanced non-clear cell renal cell cancer In **(A)** and **(B)**, PFS and OS is shown according to baseline levels of interleukin-8. In **(C)** and **(D)**, PFS is shown according to baseline levels of interleukin-13 and soluble tumor necrosis factor receptor II, respectively.

Multivariable Cox regression models identified that high levels of IL-8 (HR = 3.13; 95% CI: 1.41-6.92; p=0.005), IL-13 (HR = 3.36; 95% CI: 1.49-7.58; p=0.004), and sTNF-RII (HR = 2.21; 95% CI: 1.04-4.72; p=0.04) were the only biomarkers independently associated with shorter PFS (Figure 1A, 1C, 1D) irrespective of treatment group. In addition, high IL-8 levels were independently associated with significantly shorter OS in both treatment groups (HR = 3.55; 95% CI: 1.55-8.14; p=0.003) (Figure 1B). These associations persisted after controlling for all other established prognostic factors, including performance status, IMDC or MSKCC risk groups.

**Figure 2:**
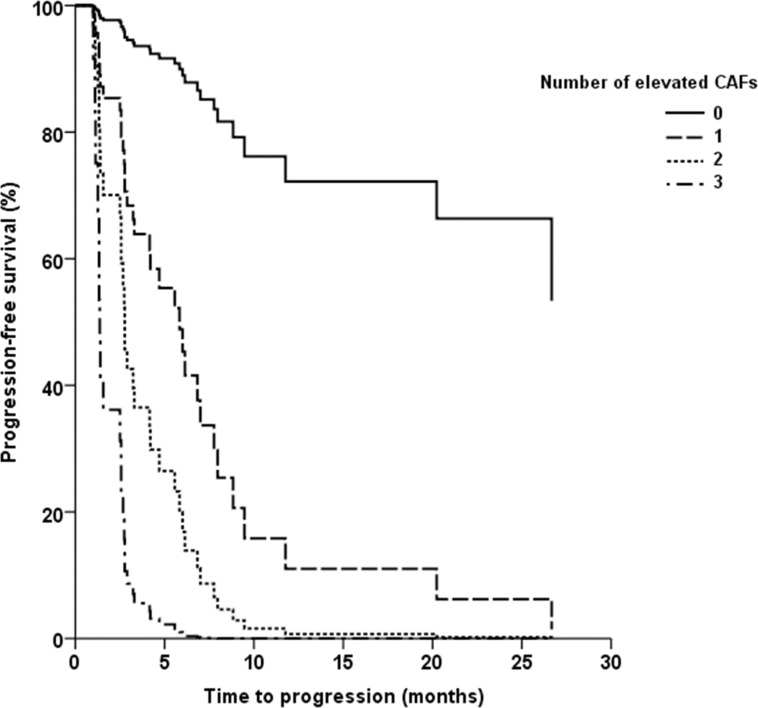
Kaplan-Meier plot of progression-free survival (PFS) according to the number of elevated circulating cytokine and angiogenic factors (IL-8, IL-13, or sTNF-RII)

Cox regression models evaluating the effect of IL-8, IL-13 or sTNF-RII on PFS (Figure 2) alone or in combination showed that patients with high levels of all three CAFs had significantly shorter PFS compared with those that had zero (HR = 43.5; 95% CI: 4.4-500; p=0.001) or only one (HR = 6.5; 95% CI: 2.1-20; p=0.001) CAFs elevated. A similar trend was seen when comparing patients with three elevated CAFs vs those with 2, although it did not reach statistical significance (HR = 2.9; 95% CI: 0.97-8.5; p=0.057). These significant associations between the number of elevated CAFs and PFS persisted after adjusting for all major clinical and laboratory variables such as age, gender, IMDC or MSKCC risk groups, performance status, or histology.

**Figure 3:**
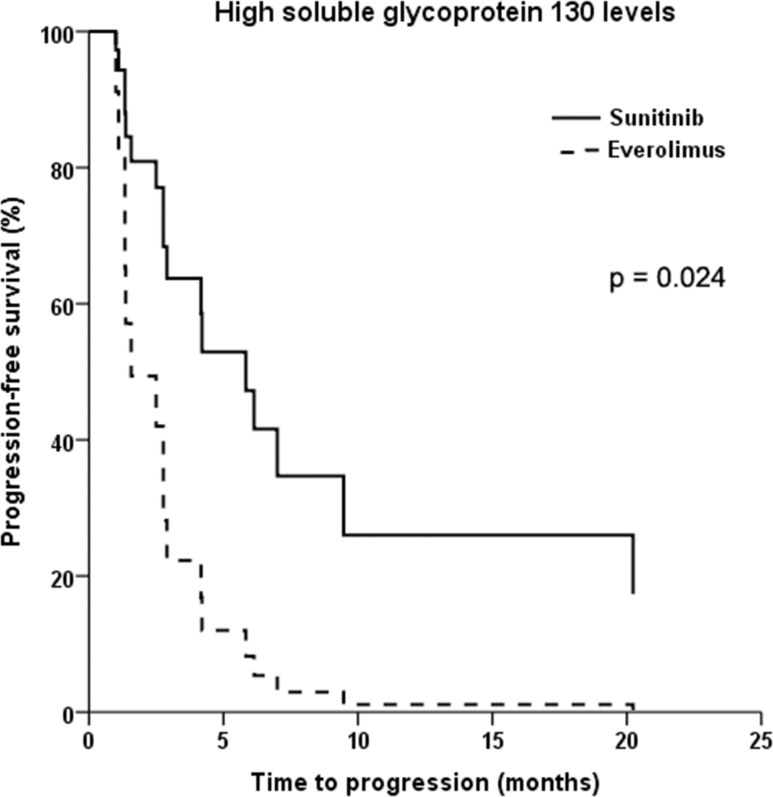
Kaplan-Meier plot of progression-free survival in patients with advanced non-clear cell renal cell cancer and high baseline levels of soluble glycoprotein 130 treated with sunitinib versus everolimus

Analyses of the two-way interaction between treatment group and each CAF showed that sgp130 was the only significant predictor of response to sunitinib compared with everolimus (p=0.031). Whereas patients with low baseline sgp130 levels had similar PFS when treated with either drug (HR = 1.28; 95% CI: 0.46-3.60; p=0.637), patients with high sgp130 levels had significantly longer PFS when treated with sunitinib compared with everolimus (HR = 0.30; 95% CI: 0.11-0.85; p=0.024) (Figure 3). This effect was independent of tumor histology. No other pretreatment CAF levels were found to be significant predictors of a differential response to therapy. The magnetic bead-based assays used to measure IL-8, sTNF-RII, and sgp130 levels showed high intra-assay precision with coefficients of variation (CV) <10% in all samples, and all samples were within the detection range. The IL-13 assay showed higher variability with CV >10% (but <20%) in 2/44 samples, as well as OOR values in 3/44 samples.

### Unsupervised hierarchical clustering by baseline CAF levels

**Figure 4:**
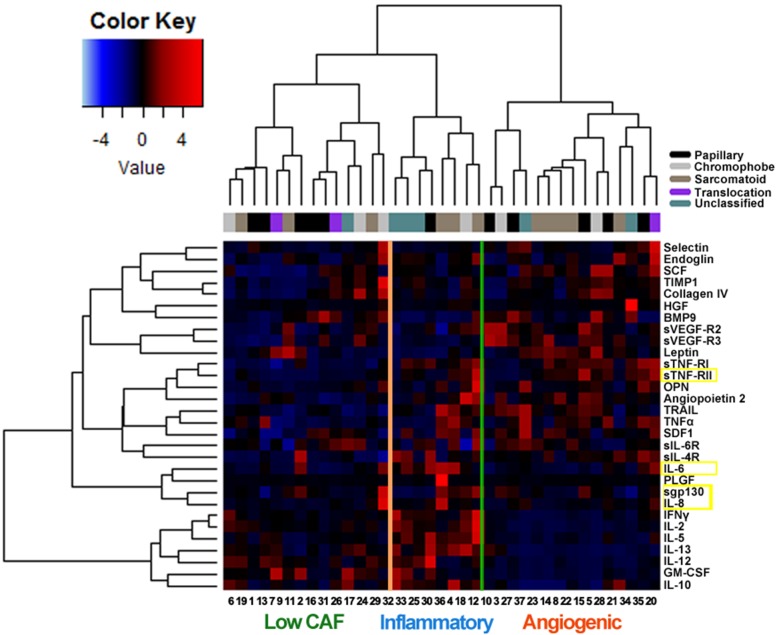
Unsupervised hierarchical cluster analysis (lower expression levels in blue, higher levels in red) of circulating cytokine and angiogenic factors (CAFs) in 37 patients with advanced non-clear cell renal cell carcinoma prior to therapy with either everolimus or sunitinib The 4 CAFs determined to be prognostic or predictive in multivariable analyses are highlighted in yellow. The histological subtype of each tumor is also shown. Three main clusters of patients are identified: the “angiogenic” group (n=15) was characterized by relatively higher levels of proangiogenic and hypoxia-associated factors such as sVEGF-R2, sVEGF-R3, TNFα and selectin. The “inflammatory” group (n=8) had higher levels of proinflammatory mediators such as IL-2, IL-5, IL-6, IL-8, IL-10, IL-12, IL-13 and IFNγ. A third cluster of patients (n=14) had relatively low expression of the CAFs defining the other two groups.

Unsupervised hierarchical clustering identified 3 main groups of patients (Figure 4). The larger group (“angiogenic group”, n=15; 4 treated with everolimus and 11 treated with sunitinib) was characterized by low levels of proinflammatory factors and relatively higher levels of proangiogenic and hypoxia-associated factors such as sVEGF-R2, sVEGF-R3, TNFα, and E-selectin. The second group (“inflammatory group”, n=8; 6 treated with everolimus and 2 treated with sunitinib) had higher levels of interleukins (IL-2, IL-5, IL-6, IL-8, IL-10, IL-12, IL-13) and other proinflammatory mediators such as IFNγ. The third cluster of patients (“low CAF group”, n=14; 6 treated with everolimus and 8 treated with sunitinib) had relatively lower concentrations of the CAFs defining the other two groups.

## DISCUSSION

Our analysis of pretreatment CAF levels in patients with nccRCC identified plasma IL-8 concentration as a prognostic marker of PFS and OS. This is consistent with our previous profiling of an independent cohort of nccRCC, which showed elevated IL-8 levels to be associated with shorter OS [7]. High IL-8 concentrations have also been associated with shorter PFS in patients with clear-cell RCC [13, 14]. These results indicate that IL-8 is a robust prognostic biomarker. IL-8 is a pro-inflammatory cytokine that facilitates tumor invasion, angiogenesis, metastasis, and resistance to therapy [15, 16]. Although we previously found that baseline levels of sTNF-RI, sVEGF-R2 and TGF-α were associated with worse survival in nccRCC [7], we were unable to replicate these results in the present study. This may reflect the significant molecular heterogeneity of this tumor group and the small number of patients.

We found that high plasma IL-13 and sTNF-RII concentrations were associated with worse PFS. IL-13 is a key regulator of type 2 helper T cells (Th2) that suppress anti-tumor immune surveillance, thus facilitating cancer invasion and metastasis [17]. sTNF-RII is the circulating form of the membrane-bound TFN-RII, which mediates TNFα signaling via different pathways compared with TNF-RI [18]. It is released into the circulation via alternative mRNA splicing leading to loss of the transmembrane and cytoplasmic domains, or via shedding of the membrane-bound form within vesicles such as exosomes [19]. The plasma concentration of sTNF-RII significantly increases with tumor stage in patients with RCC [20], and has been shown to be associated with worse outcomes in patients with advanced chronic kidney disease [21]. In conjunction with prior evidence linking sTNF-RI to poor prognosis [7], our present results provide additional evidence that TNF signaling pathways have prognostic relevance in nccRCC. These observations will need to be confirmed in other nccRCC patient cohorts.

We identified high plasma sgp130 concentrations to be predictive of a better response to sunitinib compared with everolimus. Glycoprotein 130 (gp130) is a signal-transducing receptor subunit involved in multiple inflammatory and tumorigenic pathways [22]. The soluble form of gp130 (sgp130) antagonizes the binding of IL-6 to sIL-6R and selectively abrogates IL-6 trans-signaling, including the phosphorylation of signal transducer and activator of transcription 3 (STAT3) [23–25]. Mechanistic studies have indicated that sunitinib can crosstalk with this pathway by inhibiting STAT3 in RCC tumor cells [26]. The biological mechanism underlying the association between sgp130 and sunitinib efficacy in nccRCC warrants further study, particularly since we did not find a similar correlation between plasma IL-6 or sIL-6R levels and patient outcomes in our cohort. The first study to investigate pretreatment CAFs in RCC prior to sunitinib or everolimus therapy [14] used patients enrolled in the multicenter, randomized phase II RECORD-3 trial [27]. Only a minority of these patients had nccRCC. In addition, the study profiled a different set of CAFs, which did not include sgp130 or IL-13 [14].

The VEGF pathway is a major molecular target of sunitinib, and circulating sVEGF-R2 and sVEGF-R3 may serve as biomarkers of VEGF-dependent angiogenesis and cancer growth. Although reductions in sVEGF-R2 and sVEGF-R3 levels have been shown to be associated with clinical response in patients with advanced RCC [28], baseline levels of these CAFs did not predict a better response with sunitinib compared with everolimus in our patients. This may be due to the lower dependence of nccRCC tumors on VEGF pathways.

In addition to the ESPN trial, the multicenter, open-label, randomized phase II ASPEN trial also evaluated sunitinib versus everolimus in a nccRCC cohort [10]. In contrast to ESPN, ASPEN showed improved PFS in patients treated with sunitinib compared with those treated with everolimus, except in patients who had chromophobe RCC or poor-risk disease [10]. CAF profiling can complement tumor tissue or serum DNA diagnostics and provide additional information that can guide treatment decisions in such cases. Compared with tumor tissue sampling, CAF analysis is a much less invasive procedure that requires a simple blood draw. Longitudinal CAF profiling may also identify biomarkers for acquired resistance [29]. Although serum molecular DNA analyses can provide valuable diagnostic information, protein-based assays offer additional opportunities for stratifying and monitoring disease activity because proteins are more direct mediators of functions occurring within tumor cells and their microenvironment. Several robust commercial assays are available for general use, and the ones used in the present study showed high precision. Because sample preparation and storage can affect assay performance [30], the samples used in the present study were collected and processed in a standardized manner. Further studies will be needed to assess how handling variations can affect CAF measurements by magnetic bead-based assays such as those used in the present analysis.

Histopathological and molecular profiling have shown considerable heterogeneities between nccRCCs tumors [1, 2]. The present study investigated whether there are common CAF expression patterns between these histologically and genetically distinct tumors that can be clinically exploited. Unsupervised hierarchical clustering of CAF expression in our patient cohort revealed three distinct phenotypes. One group showed relatively low CAF concentrations. Another group is characterized by an “angiogenic” signature with high levels of proangiogenic factors and lower levels of inflammatory cytokines. This group of patients demonstrates relatively higher levels of HGF and may be more likely to respond to MET inhibitors such as cabozantinib. We plan to test this hypothesis by performing baseline CAF profiling in nccRCC patients treated with cabozantinib or other c-MET inhibitors. Conversely, the “inflammatory” group of tumors demonstrated high concentrations of inflammatory mediators and relatively low expression of proangiogenic molecules. This is a similar pattern to the one we previously reported in clear-cell RCC [31]. Tumor-infiltrating lymphocytes and other immune cells may trigger their own suppression by producing inflammatory cytokines that drive the expression of immune checkpoint molecules such as programmed death-ligand 1 (PD-L1) [32]. Thus, tumors belonging to the “inflammatory” group may be susceptible to PD-L1 or programmed cell death 1 (PD-1) inhibitors, which would shift the balance of the inflammatory milieu towards anti-tumor cytotoxic immune responses.

The present study is limited by its exploratory nature and modest number of patients. Although our validation of prior findings is reassuring, the possibility of false positive associations remains. In addition, due to the lack of statistical power, we may have failed to detect clinically relevant CAF signatures. Therefore, our results will need to be independently evaluated in separate and larger patient cohorts.

In conclusion, this is the first study to specifically investigate in nccRCC the prognostic and predictive value of circulating biomarkers in patients treated with a multitargeted antiangiogenic TKI or an inhibitor of the mTOR pathway. Our findings support the use of CAF analyses to define distinct subgroups of patients with nccRCC who have a worse prognosis or will benefit less from sunitinib compared with everolimus. We identified prognostic biomarkers related to the TNF pathway (sTNF-RII) and immunomodulation (IL-8 and IL-13). In addition, mediators of IL-6 signaling such as sgp130 may affect response to therapy in these patients. If validated prospectively, these results will help optimize trial design and guide therapeutic decisions.

## MATERIALS AND METHODS

### Patients

The study design and patient population of the ESPN trial (NCT01185366) have been reported elsewhere [12]. ESPN enrolled a total of 73 patients with papillary, chromophobe, collecting duct, Xp11.2 translocation, unclassified, or clear-cell with >20% sarcomatoid transformation RCC patients who had not received prior systemic therapy. Patients were randomized 1:1 to standard dosing schedules of either sunitinib or everolimus. The primary endpoint was PFS. Secondary endpoints included OS and objective response rate (ORR). The study was approved by the institutional review board or ethics committee of each participating center and was conducted in accordance with the Declaration of Helsinki and good clinical practice guidelines.

### Collection of plasma samples and CAF analysis

All patients in this study signed informed consent for the collection of blood for biomarker profiling. Only patients with pre-treatment blood samples (44/73 patients) were included in the study. Specimens were obtained within 14 days prior to the first dose of the study drug. Peripheral venous blood for plasma preparation was collected into 8-mL sodium citrate tubes (BD, Franklin Lakes, NJ, http://www.bd.com), and stored at -80°C. Prior to CAF analysis, samples were thawed at room temperature and then centrifuged at 14000 *g* for 10 minutes at 4°C. Clear supernatant was then transferred to a labeled polypropylene tube. The concentrations of 44 CAFs were measured at the antibody-based proteomics core at Baylor College of Medicine (Houston, TX) using 7 different Luminex magnetic bead-based assays (Kit#HSTCMAG28SPMX13, HKI5MAG-99K-02, HAGP1MAG-12K-09, HCYP2MAG-62K-03, HSCRMAG-32K-07, HCVD4MAG-67K-01, and HBNMAG-51K-01; EMD Millipore, Billerica, MA, http://www.emdmillipore.com) according to the manufacturer's protocol. The CAFs that were profiled were selected based on their biological relevance to RCC, the mechanism of action of sunitinib and everolimus, commercial availability, as well as prior data indicating clinical relevance [7, 31, 33]. The plates were analyzed using the Bio-Plex 200 system (Bio-Rad, Hercules, CA, http://www.bio-rad.com). Each sample was analyzed in duplicate, and the analysis was blinded to clinical outcome. CAFs with >25% out of range (OOR) samples were omitted from the analysis. Accordingly, 30/44 CAFs (68.2%) were included in the analysis reported in this manuscript (Supplementary Table 1). For these 30 CAFs, OOR values were treated following the same convention previously established by our group [7, 31]: we replaced above-range OOR values with the highest measured value for the corresponding CAF, whereas below-range OOR values were replaced by the lowest value on the standard curve divided by half.

### Statistical analysis

The Kolmogorov-Smirnov test was used for analysis of variance of all continuous variables. The choice of methods for statistical comparisons of continuous variables was based on whether the data distribution permitted parametric or non-parametric analysis. Categorical variables were compared using Fisher's exact tests. We dichotomized values for each CAF as “low” if ≤ median and “high” if > median. This dichotomization cut point has consistently yielded optimal results and produces more equally balanced subgroups [7, 31, 33, 34]]. To determine which CAFs were independently associated with PFS and OS, we constructed multivariable Cox regression models using a fully stepwise selection algorithm, based on likelihood ratio tests, that required covariables to have a p value <0.1 for entry (to increase sensitivity) and <0.05 for retention in the model. Candidate variables included the 30 CAFs as well as age, race, gender, prior nephrectomy, performance status, international metastatic renal cell carcinoma database consortium (IMDC) or Memorial Sloan Kettering Cancer Center (MSKCC) risk group, tumor histology, and treatment group. Log-minus-log survival plots were constructed to confirm the proportional hazard assumption.

To identify variables that were associated with a different response to sunitinib versus everolimus, we constructed additional Cox proportional hazards models testing the two-way interaction terms of CAFs with treatment group, using PFS as the outcome. To identify distinct CAF-based molecular phenotypes among nccRCC patients, we performed unsupervised hierarchical clustering using Ward's method (“ward.D2” in R) and Pearson's correlation coefficient as a dissimilarity measure. For this analysis, CAF values were standardized to a mean of zero and standard deviation of one.

A p value of <0.05 was considered statistically significant unless otherwise specified. Given the exploratory nature of this study, no adjustment for multiple comparisons was made based on our prespecified analysis plan. Unless otherwise indicated, all statistical analyses were completed using SPSS 23.0 (IBM Corp, Armonk, NY) and R (Foundation for Statistical Computing, Vienna, Austria) [35].

## SUPPLEMENTARY MATERIALS FIGURES AND TABLES





## Abbreviations

BMP9: bone morphogenetic protein 9
CAF: cytokine and angiogenic factor
GM-CSF: granulocyte-macrophage colony-stimulating factor
HGF: hepatocyte growth factor
HR: hazard ratio
IFNγ: interferon gamma
IL-10: interleukin-10
IL-12: interleukin-12
IL-13: interleukin-13
IL-5: interleukin-5
IL-6: interleukin-6
IL-8: interleukin-8
IMDC: international metastatic renal cell carcinoma database consortium
MSKCC: Memorial Sloan Kettering Cancer Center
mTOR: mechanistic target of rapamycin
nccRCC: non-clear cell renal cell carcinoma
OOR: out of range
OS: overall survival
PD-1: programmed cell death 1
PD-L1: programmed death-ligand 1
PFS: progression-free survival
PLGF: placental growth factor
RCC: renal cell carcinoma
SCF: stem cell factor
SDF1: stromal cell-derived factor 1
sgp130: soluble glycoprotein 130
sIL-4R: soluble IL-4 receptor
sIL-6R: soluble IL-6 receptor
sTNF-RI: soluble tumor necrosis factor receptor I
sTNF-RII: soluble tumor necrosis factor receptor II
sVEGF-R2: soluble vascular endothelial growth factor receptor-2
sVEGF-R3: soluble vascular endothelial growth factor receptor-3
TIMP1: tissue inhibitor of metalloproteinase 1
TKI: tyrosine kinase inhibitor
TRAIL: tumor necrosis factor-related apoptosis-inducing ligand
TNFα: tumor necrosis factor alpha
VEGF: vascular endothelial growth factor
VHL: von Hippel-Lindau
