# Supplementary material for: Plasma cytokine and angiogenic factors associated with prognosis and therapeutic response to sunitinib vs everolimus in advanced non-clear cell renal cell carcinoma
Source: Oncotarget. 2017 Feb 2;8(26):42149–58. doi: 10.18632/oncotarget.15011 (PMC5522056; doi:10.18632/oncotarget.15011)
Supplement: Supplementary file 1 [file oncotarget-08-42149-s001.pdf]

Plasma cytokine and angiogenic factors associated with prognosis and therapeutic response to sunitinib vs everolimus in advanced non-clear cell renal cell carcinoma

SUPPLEMENTARY MATERIALS

Supplementary Table 1: Median CAF levels in pg/ml (interquartile range in parentheses) of each treatment group.

See Supplementary File 1
