# Supplementary material for: Plasma cytokine and angiogenic factors associated with prognosis and therapeutic response to sunitinib vs everolimus in advanced non-clear cell renal cell carcinoma
Source: Oncotarget. 2017 Feb 2;8(26):42149–58. doi: 10.18632/oncotarget.15011 (PMC5522056; doi:10.18632/oncotarget.15011)
Supplement: Supplementary file 2 [file oncotarget-08-42149-s002.docx]

**Supplementary Table 1:** **Median CAF levels in pg/ml (interquartile range in parentheses) of each treatment group**

**Everolimus (n=16)**

**Sunitinib (n=21)**

**p value^a^**

GM-CSF

16.38 (12.0 - 30.42)

22.81 (11.77 - 41.94)

0.783

IFNγ

5.75 (2.27 - 9.57)

4.20 (2.76 - 6.60)

0.408

IL-10

6.27 (3.12 - 9.01)

4.79 (3.24 - 8.64)

0.613

IL-12

2.20 (0.86 - 3.98)

1.31 (0.52 - 1.89)

0.128

IL-13

4.64 (2.53 - 9.40)

1.66 (0.92 - 5.80)

0.05

IL-2

2.48 (0.80 - 3.04)

1.23 (0.64 - 1.55)

0.089

IL-5

1.42 (0.33 - 2.76)

1.06 (0.63 - 1.50)

0.326

IL-6

1.66 (0.75 - 2.26)

1.14 (0.70 - 2.35)

0.391

IL-8

5.18 (3.93 - 7.82)

3.70 (2.74 - 5.89)

0.172

TNFα^c^

4.97 (2.91 - 6.60)

3.33 (2.21 - 5.41)

0.114

TIMP1

161.94 (99.88 - 230.99)

183.08 (48.39 - 224.58)

0.818

Collagen IV

485.88 (298.65 - 645.33)

512.69 (192.96 - 610.26)

0.927

Angiopoietin-2

2348.86 (1649.04 - 3287.09)

1852.58 (1324.62 - 2452.29)

0.086

BMP9

20.43 (11.0 - 64.98)

31.18 (16.61 - 48.23)

0.668

Endoglin

837.37 (741.07 - 978.80)

741.06 (374.64 - 985.23)

0.118

HGF

132.39 (108.23 - 283.57)

128.12 (88.28 - 167.48)

0.172

Leptin

13499.30 (4723.49 - 26140.26)

17133.43 (10283.26 - 42583.34)

0.257

PLGF

3.27 (0.35 - 7.40)

5.20 (2.07 - 8.12)

0.242

SCF

26.64 (16.71 - 34.94)

44.48 (32.40 - 52.13)

0.041

TRAIL

61.46 (47.15 - 75.40)

51.95 (39.77 - 70.59)

0.602

SDF1

3659.83 (2734.29 - 5282.59)

4323.95 (3426.99 - 5385.91)

0.581

sgp130

132704.03 (115407.13 - 154382.26)

130346 (122895.97 - 145221.90)

0.902

sIL-4R

3168.76 (2847.12 - 3503.85)

3268.77 (2961.48 - 3761.52)

0.520

sIL-6R

20151.48 (19114.58 - 24728.67)

21169.00 (15101.10 - 25222.23)

0.806

sTNF-RI

2258.38 (1506.39 - 2846.51)

1819.44 (1610.64 - 2873.07)

0.168

sTNF-RII

8877.92 (6150.42 - 11579.76)

6270.38 (5532.98 - 9965.96)

0.462

sVEGF-R2

13646.02 (10452.55 - 16838.84)

14327.65 (12693.05 - 16604.89)

0.032

sVEGF-R3

3281.26 (1949.95 - 3929.40)

5698.14 (3023.71 - 7383.72)

0.581

Selectin

61.28 (36.61 - 77.98)

48.18 (39.92 - 66.59)

0.646

Osteoprotegerin

55997.88 (33657.26 - 109379.78)

52380.43 (36849.24 - 70279.00)

0.365

^a^Mann–Whitney U test

BMP9: bone morphogenetic protein 9; ECOG: Eastern Cooperative Oncology Group; GM-CSF: granulocyte-macrophage colony-stimulating factor; HGF: hepatocyte growth factor; IFNγ: interferon gamma; IL-10: interleukin-10; IL-12: interleukin-12; IL-13: interleukin-13; IL-5: interleukin-5; IL-6: interleukin-6; IL-8: interleukin-8; PLGF: placental growth factor; SCF: stem cell factor; SDF1: stromal cell-derived factor 1; sgp130: soluble glycoprotein 130; sIL-4R: soluble IL-4 receptor; IMDC: international metastatic renal cell carcinoma database consortium; MSKCC: Memorial Sloan Kettering Cancer Center; sIL-6R: soluble IL-6 receptor; sTNF-RI: soluble tumor necrosis factor receptor I; sTNF-RII: soluble tumor necrosis factor receptor II; sVEGF-R2: soluble vascular endothelial growth factor receptor-2; sVEGF-R3: soluble vascular endothelial growth factor receptor-3; TIMP1: tissue inhibitor of metalloproteinase 1; TRAIL: tumor necrosis factor-related apoptosis-inducing ligand; TNFα: tumor necrosis factor alpha.
